# Sexual, Physical, and Emotional Maltreatment in Childhood Are Differentially Associated With Sexual and Physical Revictimization in Adulthood

**DOI:** 10.1177/08862605221111411

**Published:** 2022-07-22

**Authors:** Jessica Rowe, Jasmine Chananna, Simone Cunningham, Kate L. Harkness

**Affiliations:** 1 Queen’s University, Kingston, Ontario, Canada; 2 East Michigan University, MI, USA

**Keywords:** sexual abuse, child abuse, physical abuse, child abuse, sexual assault, GLBT, revictimization, sexual assault

## Abstract

Childhood maltreatment increases risk for sexual and physical revictimization in adulthood. The goal of the current study was to examine whether this risk is associated with specific maltreatment types (i.e., sexual vs. physical vs. emotional maltreatment vs. neglect) and perpetrators (i.e., mother vs. father). Participants included 720 adult women from North America and the United Kingdom, recruited through the online platform Prolific Academic. The severity of childhood maltreatment and adult physical and sexual victimization were assessed in two separate sessions through self-report questionnaires. All maltreatment types were modeled together to account for their co-occurrence. Greater severity of sexual maltreatment was significantly and independently associated with greater risk for sexual, physical, and sexual + physical revictimization. Further, in the full sample, risk of revictimization was predicted by greater severity of father-perpetrated emotional and physical maltreatment. In contrast, in subgroup analyses focusing on plurisexual (i.e., bi/pansexual) women, risk of revictimization was predicted by greater severity of mother-perpetrated emotional and physical maltreatment. These results suggest that girls with sexual and emotional maltreatment histories are at highest risk for revictimization. Future research identifying the biological, psychological, and social sequelae of these specific exposures may enable the development of specific intervention programs that have the potential for maximum efficacy in preventing further violence against women most at risk.

Globally, it has been estimated that half of all children, per year, are victims of sexual, physical, and/or emotional maltreatment (Hillis et al., 2016). Not only is childhood maltreatment extremely prevalent worldwide, it is also associated with significant societal cost, with annual economic burden estimates ranging from $428 billion to $2 trillion in the United States alone (Peterson et al., 2018). Exposure to maltreatment in childhood predicts a wide range of negative outcomes, including lower educational and occupational attainment (Romano et al., 2015), poorer mental health (Kessler et al., 2010), and lower overall quality of life (Corso et al., 2008).

Notably, maltreatment in childhood significantly raises the risk of both sexual and physical revictimization throughout adolescence and adulthood (Walker et al., 2017; Widom et al., 2008). There is evidence that the relationship between childhood maltreatment and sexual and physical revictimization is stronger in women than in men(Karsberg et al., 2019) and women who experience victimization in both childhood and adulthood are at a greater risk for poor outcomes than those who were victimized only in childhood or adulthood (Classen et al., 2005; Kimerling et al., 2007). While the general association of childhood maltreatment to risk of adult sexual and physical revictimization has been established, what is far less well understood is whether this risk is *differentially* associated with particular types of maltreatment (i.e., sexual vs. physical vs. emotional maltreatment perpetrated by the mother vs. father figure). Further, almost no research has sought to examine whether there are *differential links* between childhood maltreatment and revictimization for women in different types of adult relationship contexts (i.e., monosexual vs. plurisexual women).This fine-grained approach has the potential to broaden our understanding of the relation between childhood maltreatment and later victimization and inform early identification of women at risk for violent outcomes.

## Childhood Maltreatment and Sexual Revictimization

Freyd’s betrayal trauma theory proposes that childhood trauma high in betrayal—defined as sexual, emotional, or physical maltreatment perpetrated by someone close to the victim, such as a parent—puts survivors at especially high risk for later revictimization (Freyd et al., 2007; Gobin & Freyd, 2009). Betrayal theory proposes that, while potentially adaptive in the abusive childhood context, in adulthood the “scars” left by maltreatment raise women’s likelihood of being exposed to environments in which violence is likely to reoccur. These scars can be cognitive (e.g., core beliefs of abandonment and dependency; Zheng et al., 2022), emotional (e.g., enduring guilt and shame; Freyd et al., 2007), and even neurobiological (e.g., neurohormonal, epigenetic, and functional/structural changes; Nemeroff, 2016). Together, such negative sequelae of maltreatment are theorized to promote maladaptive behaviors, such as drug use and risky sexual behavior, which raise risk for sexual revictimization (Gobin & Freyd, 2009).

The empirical evidence is largely supportive of betrayal trauma theory (see Arata, 2002),with longitudinal studies suggesting that half of the two-thirds of girls who are sexually maltreated in childhood or adolescence will be sexually revictimized in adulthood (Classen et al., 2005; Walker et al., 2017). However, empirical evidence regarding the *differential* association of sexual maltreatment *over* other forms of maltreatment to risk for sexual revictimization is mixed. In a retrospective study of 7,272 adults, Ports et al. (2016) found that histories of childhood sexual, physical, and emotional maltreatment, assessed with the Adverse Childhood Experiences Scale, were each significantly associated with adult sexual revictimization, even when modeled simultaneously. Sexual maltreatment was associated with the largest effect size, followed by emotional maltreatment. Similarly, in a retrospective study of 11,056 women, Kimerling et al. (2007) found that sexual maltreatment was associated to adult sexual revictimization with a larger effect size (Odd Ratio [OR] = 4.3) than physical maltreatment (OR = 2.5).

In direct contrast, in a study of 8,000 women that assessed maltreatment through retrospective self-report, Desai et al. (2002) found that childhood *physical* maltreatment (OR = 2.7) had a descriptively stronger relation to adult sexual victimization than childhood sexual maltreatment (OR = 1.8). Further, in a study of women with major depressive disorder, Cunningham et al. (2019) reported that when childhood physical, sexual, and emotional maltreatment were modeled simultaneously, only *emotional* maltreatment was significantly independently associated with sexual revictimization in adolescence. A particular strength of this latter study was its use of a contextual interview assessment that bases ratings of maltreatment severity on contextual features such as frequency, duration, and relationship to perpetrator. Similarly, Pezzoli et al. (2020) found in a sample of 8,376 Finnish women that childhood emotional maltreatment was significantly more strongly associated with adult sexual victimization than were childhood sexual or physical maltreatment.

In summary, research to date suggests that sexual, physical, and emotional maltreatment in childhood elevate risk for later sexual revictimization. However, studies using strong, contextual methods to assess childhood maltreatment appear to implicate emotional maltreatment more strongly than sexual or physical maltreatment.

## Childhood Maltreatment and Physical Revictimization

Researchers have appealed to Bandura’s (1977) social learning theory to suggest that those who have experienced physical violence in childhood maybe more likely than those without this history to accept interpersonal violence by others in adulthood (Schuster & Tomaszewska, 2021). And, the evidence generally appears to support a stronger relation of childhood physical maltreatment, over sexual or emotional maltreatment, to later physical revictimization. For example, in Desai et al.’s (2002) report, childhood physical maltreatment was associated with a larger effect size (OR = 3.4) in relation to adult physical revictimization than childhood sexual maltreatment (OR = 2.2). Similarly, in a prospective cohort study of children with documented maltreatment, Widom et al. (2014) found that physical maltreatment (OR = 2.52), followed by neglect (OR = 1.64), had the largest effect sizes in predicting physical revictimization by a partner in adulthood (see also Widom et al., 2008). Further, in a sample of homeless adults, Edalati et al. (2016) found that only childhood physical maltreatment was significantly associated with adult physical revictimization when childhood physical, sexual, and emotional maltreatment were modeled simultaneously.

## Maternal- Versus Paternal-Perpetrated Maltreatment

Bowlby’s attachment theory (1979) proposes that there are differential patterns of development based on mother−daughter and father−daughter interactions, and suggests that young girls’ interactions with their fathers may be particularly formative in the development of *heterosexual* dating scripts. For instance, self-reports of lower paternal warmth is associated in women with lower sexual refusal self-efficacy, greater acceptance of male-partner dominance, and lower self-worth (Katz & van der Kloet, 2010). Further, harsh parenting from the father predicts early sexual debut and tendency to engage in risky sexual behavior (DelPriore et al., 2018; Ellis et al., 2012), both of which are associated with risk for sexual revictimization.

To our knowledge, only two studies have specifically compared paternal- versus maternal-perpetrated maltreatment as predictors of sexual or physical revictimization and, thus, this is an area where more research is sorely needed. In a study of women, all of whom were in an episode of depression, Cunningham et al. (2019) found that emotional maltreatment perpetrated by the father was significantly associated with sexual revictimization outside the home in adolescence, whereas emotional maltreatment perpetrated by the mother was not. Similarly, in the Pacific Island Families Study, a history of physical maltreatment perpetrated by the father was significantly associated with physical victimization in adult intimate partner relationships, whereas maltreatment perpetrated by the mother was not (Paterson et al., 2007). Therefore, an important goal of the current study is to replicate these findings in a demographically diverse sample of women not suffering from severe psychopathology, and to extend them to victimization in adulthood not solely limited to intimate partners. We predict that, father-perpetrated maltreatment will confer greater risk for revictimization compared to mother-perpetrated maltreatment.

## Sexual Revictimization and Plurisexuality

There is consistent evidence that risk for adulthood sexual victimization, specifically, is higher among women who identify as plurisexual (i.e., sexual attraction to more than one gender, including women identifying as bisexual or pansexual) than those who identify as monosexual (i.e., sexual attraction to only one gender, including women identifying as gay/lesbian or straight) (Hequembourg et al., 2013). Despite their heightened risk, plurisexual women are often either excluded from research examining risk factors for sexual revictimization or are not considered separately from monosexual women. Therefore, to our knowledge the current study is the first to examine whether the types of childhood maltreatment that raise risk for sexual revictimization in plurisexual women differ from those identified in monosexual women.

There is some tentative evidence from the literature to suggest that emotional maltreatment may be a particularly potent vulnerability factor for plurisexual women. In general, sexual minority adults report greater histories of parental rejection and criticism than nonminority adults (Fish et al., 2020), the effects of which may then be magnified by an ongoing context of societal rejection of their sexual identity. While a direct link between parental emotional maltreatment and adult revictimization has not been examined in sexual minority individuals, there is evidence that emotional maltreatment is associated in this group with heightened risk of alcohol abuse, a strong risk factor for sexual revictimization (Fish et al., 2020). Further, in plurisexual women verbal abuse from micro- (e.g., parental) and macro- (e.g., society) contexts is more likely than in monosexual women to include charges of hypersexuality and blaming for sexual violence, again raising risk for sexual victimization (Flanders et al., 2019).

As noted above, the literature in women, in general, has so far suggested that emotional maltreatment from the father may be more strongly associated with later victimization than that perpetrated by the mother. However, this literature, which does not consider women’s sexuality, has to date been interpreted with reference to the impact of father’s abuse on *heterosexual* dating scripts that then increase risk for revictimization. Therefore, a particularly novel question in the current study is which of father- versus mother-perpetrated maltreatment is a stronger predictor of revictimization in women whose later sexual partners may be of any gender.

## The Current Study

The first goal of the current study was to investigate the differential associations of specific types of childhood maltreatment to sexual and physical revictimization in adulthood in a cross-sectional, retrospective sample of adult women. This study includes the broadest range of maltreatment experiences to date, and in a particularly novel extension of the literature, we examine the differential effects of father versus mother emotional and physical maltreatment on both sexual *and* physical victimization. We hypothesize that when modeled with all other maltreatment variables, sexual maltreatment by any perpetrator, as well as emotional maltreatment or neglect perpetrated by the father, will be significantly and independently associated with sexual revictimization in adulthood. Further, we hypothesize that physical maltreatment perpetrated by the father will be significantly and independently associated with physical revictimization in adulthood.

Our second goal was to provide a novel examination of the relation between childhood maltreatment and sexual revictimization in women identifying as plurisexual. We hypothesize that sexual maltreatment and emotional maltreatment will be significantly and independently associated with adult sexual revictimization. There is currently no past research or theory to motivate hypotheses regarding the differential impact of maternal- versus paternal-perpetrated emotional maltreatment and, thus, this specific comparison is exploratory.

## Methods

### Participants

Participants included 720 women recruited through the online research platform *Prolific Academic*. Online recruitment was chosen to maximize our ability to recruit a large and demographically diverse sample of women. We chose Prolific Academic over more well-known platforms, such as MTurk, as it is specifically designed for academic research applications. Participants recruited through Prolific Academic have been shown to have higher data quality in terms of comprehension, attention, and honesty than other platforms (Peer et al., 2021). The General Research Ethics Board at Queen’s University provided ethical approval for this study. Inclusion criteria were (1) cisgender or transgender identifying women and (2) resident of Canada, the United States, or the United Kingdom. Participants were drawn from an initial sample of 1,000 women who completed an online session to screen for sexual and physical victimization in adulthood. Of this initial 1,000, 68 were excluded due to missing data or random responding. Of the remaining 932, 567 reported a history of sexual and/or physical victimization in adulthood and, thus, were invited to a second online session. An additional 233 of those who did not report adult victimization were invited to serve as a comparison group. Of the 800 who were invited to participate in the second online session, 34 chose not to participate and 9 participants were excluded due to missing data or random responding. Finally, 37 participants were excluded because they experienced chronic victimization from childhood to adulthood by the same perpetrator rather than a new perpetrator (see below).

### Measures

In addition to the questionnaires below, the online surveys included two validity items introduced at equally spaced intervals (e.g., “For this question please select strongly disagree”). Participants who failed the validity items were excluded from analyses. Further, at the end of each session was an open text question that asked participants to briefly describe what the study was about. We visually inspected each response and excluded evidence of random responding (e.g., selecting different ages at the two times of assessment). These data quality measures were implemented based on the recommendations provided by Hauser et al. (2019).

#### Demographic questionnaire

A structured questionnaire developed for the purposes of the current study included items querying gender, sexual orientation, age, ethnicity, and marital, education, and occupation status.

#### Childhood Experience of Care and Abuse Questionnaire

The Childhood Experience of Care and Abuse Questionnaire (CECA-Q) is a 50-item questionnaire that assesses maltreatment exposure prior to age 17 (Bifulco et al., 2005). The following seven maltreatment scales were examined in the current study: (1) maternal and (2) paternal emotional maltreatment; (3) maternal and (4) paternal neglect; (5) maternal and (6) paternal physical maltreatment; and (7) sexual maltreatment by any perpetrator.

The emotional maltreatment and neglect scales each included eight items rated on a scale from “1—no, not at all” to “5—yes, definitely” (⍺ = .88–.92). Items were summed for each parent on each scale (range = 8–40). Emotional maltreatment is defined as “hostility, coldness, or rejection shown to the child by parent figures” (Bifulco et al., 2005, p. 567; e.g., “She [or he] was very critical of me,” “she [or he] made me feel unwanted,” “she [or he] was very difficult to please”).

Neglect is defined as “parent’s disinterest in material care (feeding and clothing), health, schoolwork, and friendships” (Bifulco et al., 2005, p. 567; e.g., “she [or he] cared for me when I was ill” [reverse-scored], “she [or he] was interested in who my friends were” [reverse-scored], “she [or he] neglected my basic needs [food, clothing, etc.]”).

The physical maltreatment scale included four items querying the frequency and severity of violence, including the presence of injury and if the perpetrator’s anger was out of control. Items were summed separately for each parent. The sexual maltreatment scale included seven items querying the frequency, extent of perpetration (e.g., touching, intercourse), and relation to the perpetrator (e.g., relative, household member). Items were summed separately for the first and worst experiences in childhood. The greater of the two sums was used in analyses.

Population norms for the CECA-Q are not available. However, in the initial validation study of the measure, Bifulco et al. (2005) determined that scores ≥25 indicated “severe” levels of emotional maltreatment; scores ≥22 (maternal) or ≥24 (paternal) indicated “severe” neglect; and scores ≥1 indicated “severe” physical or sexual maltreatment. Means across revictimization groups in the current sample are given in Table 2.

##### Sexual and Physical Assault Scale

The Sexual and Physical Assault (SAPA) scale was created for the current study to enable an assessment of both sexual and physical victimization in adulthood (age ≥ 17) within the same scale (see Supplemental Appendix A). The sexual revictimization subscale (⍺ = .83) included six potential exposures and the physical revictimization subscale (⍺ = .74) included seven potential exposures. For each exposure, participants indicated the perpetrator(s) (e.g., “spouse,” “close family member (parent, sibling),” “stranger”) and frequency (e.g., “once only,” “2–4 times”). We subsequently used this information to contextually define the presence versus absence of “severe” sexual and/or physical revictimization. Determinations of severe revictimization were made using the criteria developed by Bifulco et al. (1994) to contextually define severe childhood physical and sexual maltreatment in their original CECA-Q interview, and with reference to the CECA-Q manual (Bifulco et al., 1994).

Five decades of research with the CECA-Q and other related stress measures (the Life Events and Difficulties Schedule; Brown & Harris, 1978) have provided strong and consistent evidence that “severely threatening” exposures are stronger predictors of health and other outcomes than are more minor exposures (see Vrshek-Schallhorn et al., 2020). Thus, we operationalized our outcome as the presence versus absence of “severe” revictimization so that it represents a construct that has maximum clinical significance and prognostic import. Constraining our focus in this way also serves to maximize the power of our statistical models (i.e., reduced variability). Thus, participants who either did not report any sexual/physical revictimization, or who reported revictimization that did not meet the criteria for “severe” (see below), were categorized as “no *severe* sexual/physical revictimization.” For the purposes of analyses, we created a “severe revictimization” variable with four levels: (1) no severe physical or severe sexual revictimization (*n* = 349), (2) severe sexual revictimization but no severe physical revictimization (*n* = 234), (3) severe physical revictimization but no severe sexual revictimization (*n* = 51), or (4) severe sexual + physical revictimization (*n* = 86).

Criteria for severe sexual victimization were met if participants endorsed at least one of the following: (a) “kissing and/or petting” *plus* endorsement of an “authority figure (e.g., doctor, priest/rabbi, teacher)” as the perpetrator regardless of frequency; (b) “kissing and/or petting” *plus* endorsement of a frequency of “2–4 times” or higher regardless of perpetrator; (c) “touching your genitals” or “touching the other person’s genitals” or “vaginal, oral, or anal intercourse” regardless of perpetrator or frequency; or (d) “verbal threats toward you or someone else” or “physical force or violence” regardless of perpetrator, frequency, or nature of victimization. Criteria for severe physical victimization were met if participants endorsed at least one of the following exposures: (a) “punching or kicking the arms, legs, or body” *plus* “bruising, black eyes, or surface cuts”; (b) “punching or kicking the head or face”; (c) “hitting with an object (e.g., belt, stick)”; (d) “threatening with a knife, gun, or other object”; or (e)“broken bones, concussion, or other serious injury (e.g., burns, deep wounds)” (regardless of where/how the participant was hit).

When examining the relation of childhood to adult victimization, it is crucial to ensure that participants are reporting on independent incidents. Reporting, instead, on the same incident that *persists* from childhood to adulthood would result in spurious associations between these two constructs. To ensure independence, we visually inspected each individual record and excluded participants (*n* = 37) whose reports of victimization on the SAPA had an age of onset prior to age 17 *and* included the same perpetrator as that reported on the CECA-Q. Further, to check the validity of our operationalization of severe victimization, we recalculated our outcome variable so that it represented the distinction between no victimization versus *any* victimization (whether severe or non-severe). The pattern of results reported here did not change, but the effect sizes were smaller, consistent with reduced power.

### Procedure

All participants took part in two online sessions 2 weeks apart hosted by the online research platform Prolific Academic. Session 1 included the SAPA and demographic questionnaire. Session 2 included the CECA-Q and a readministration of the demographic questionnaire to serve as a validity check. The study measures were presented in separate sessions for two reasons. First, we had concerns of potential low base rates of physical and/or sexual victimization in adulthood in the population of women participating in Prolific Academic studies. Therefore, as noted earlier, we over-sampled in Session 1 and chose our final sample based on participants’ endorsements of physical and/or sexual victimization on the SAPA. Second, we did not want responses on the SAPA to bias responses on the CECA-Q, or vice versa, which could potentially lead to spurious associations. Therefore, assessing them in completely separate Prolific Academic “studies,” separated in time, obscured the overall study purpose. In both the sessions, potential participants saw the study title as well as a brief description of the study. If they chose to participate, they were taken to a Qualtrics survey. In both the sessions, participants first saw a Letter of Information and provided informed consent. Participants then completed the relevant questionnaires. Each session was followed by a list of national hotlines and resources for victims of violence. Following the second session, participants also received a debriefing form that fully explained the nature of the study. After the first session, participants received $4.00 and they received $5.25 after the second because it took longer duration to complete. Both of these monetary values were at the upper end of the compensation schedule recommended by Prolific Academic.

### Data Analysis

Statistical analyses were conducted with IBM SPSS Statistics for Macintosh, Version 26.0. Preliminary chi-square analyses and one-way Analyses of Variance were conducted to examine the univariate relations between severe revictimization, the demographic characteristics of the sample, and scores on the childhood maltreatment variables.

Our first research question in the full sample was assessed with a multinomial logistic regression. The seven childhood maltreatment variables were standardized and entered as a single block. This approach tests whether the variance accounted for in the revictimization outcomes by each maltreatment variable is significant over and above the variance accounted for by each of the other maltreatment variables. By standardizing the coefficients, the relative strength of each of the maltreatment types can be compared. Severe revictimization was the dependent variable. Three indicator contrasts were specified to compare the no severe victimization group (indicator) to the (1) severe sexual victimization only group, (2) severe physical victimization only group, and (3) severe sexual + physical victimization group. Our second research question examining risk for sexual revictimization in women identifying as plurisexual was tested with a multivariate general linear model. This approach again assesses the multivariate associations of the maltreatment types to revictimization, thereby accounting for their overlap.

## Results

### Preliminary Demographic Associations

The four revictimization groups did not differ significantly in ethnicity, marital, or occupation status (see Table 1). However, the no severe victimization group was significantly younger than the severe physical revictimization (contrast = 4.63, *p* = .01) and severe sexual + physical revictimization (contrast = 3.42, *p* = .02) groups. Further, having high school credit or lower was associated with an increased risk of sexual (contrast = 0.10, *p* = .001), physical (contrast = 0.11, *p* = .001), and sexual + physical revictimization (contrast = 0.12, *p* = .004). Revictimization also differed across sexual identities. Follow-up indicator contrasts showed that those who identified as plurisexual had higher rates of sexual only (contrast = 0.07, *p* = .01) and sexual + physical revictimization (contrast = 0.12, *p* = .002) compared to those who identified as monosexual. The multinominal logistic regression models here were robust to the inclusion of demographic covariates. Thus, for ease of interpretation, models without the inclusion of covariates are presented here.

**Table 1.** Demographic Characteristics of the Sample Stratified by Severe Revictimization Group.

	None (*n* = 349)	Sexual only (*n* = 234)	Physical only (*n* = 51)	Sexual + physical (*n* *=* 86)	*F/*χ^2^
Age, *M* (SD)	32.58 (12.13)	34.27 (12.37)	37.16 (13.01)	36.02 (11.46)	3.50*
Gender, *n* (%)					4.29
Cisgender	347 (99.4)	234 (100)	51 (100)	85 (98.8)	
Transgender	2 (0.6)	0 (0)	0 (0)	1 (0.2)	
Ethnicity, *n* (%)					19.12
White	250 (71.6)	183 (78.2)	36 (70.5)	71 (82.5)	
Black	23 (6.5)	15 (6.4)	7 (13.7)	5 (5.8)	
Asian	50 (14.3)	18 (7.7)	6 (11.8)	3 (3.5)	
Hispanic	5 (1.4)	7 (3.0)	1 (2.0)	2 (2.3)	
Mixed/other	21 (6.0)	11 (4.7)	1 (2.0)	5 (5.8)	
Sexual orientation					14.73*
Monosexual	311 (89.1)	192 (82.0)	47 (92.2)	66 (76.7)	
Plurisexual	33 (9.4)	39 (16.7)	4 (7.8)	19 (22.1)	
Other/not specified	5 (1.4)	3 (1.3)	0 (0.0)	1 (0.0)	
Marital status					11.72
Never married	175 (50.1)	108 (46.2)	23 (45.1)	41 (47.7)	
Married/partnership	148 (42.4)	98 (41.8)	20 (39.2)	33 (38.3)	
Separated	6 (1.7)	5 (2.1)	1 (2.0)	5 (5.8)	
Divorced	16 (4.6)	20 (8.5)	6 (11.7)	6 (7.0)	
Widowed	4 (1.1)	3 (1.3)	1 (2.0)	1 (1.2)	
Education, *n* (%)					20.97**
High school or lower	71 (20.3)	25 (10.7)	5 (9.8)	7 (8.1)	
Some postsecondary	72 (20.6)	41 (17.5)	11 (21.6)	25 (29.1)	
Postsecondary	206 (59.0)	168 (71.8)	35 (68.6)	54 (62.8)	
Employment, *n* (%)					3.91
Employed/student	269 (77.1)	173 (73.9)	33 (64.7)	63 (73.3)	
Not employed	80 (22.9)	61 (26.1)	18 (35.3)	23 (26.7)	

* *p* < .05. **p < .01.

### Multivariate Relations of Childhood Maltreatment Types to Adulthood Revictimization

Means and standard deviations of childhood maltreatment across revictimization groups, as well as intercorrelations among the maltreatment types, are given in Table 2. As expected, all the maltreatment variables were significantly intercorrelated (see Higgins & McCabe, 2001), which emphasizes the importance of accounting for this overlap statistically.

**Table 2.** Means and Standard Deviations Across Severe Revictimization Groups and InterCorrelations of Maltreatment Variables.

Variable	None	Sexual only	Physical only	Sexual + physical	1	2	3	4	5	6
1. Maternal emotional maltreatment	16.64 (8.03)	19.06 (8.37)	17.82 (8.39)	21.65 (9.48)	1.00					
2. Paternal emotional maltreatment	16.81 (7.76)	19.16 (8.85)	17.65 (8.44)	21.64 (9.53)	.45*	1.00				
3. Maternal neglect	14.07 (6.04)	16.06 (6.84)	14.96 (5.94)	18.51 (8.22)	.78*	.46*	1.00			
4. Paternal neglect	18.77 (7.68)	19.89 (7.68)	19.65 (8.60)	22.38 (8.78)	.43*	.75*	.55*	1.00		
5. Maternal physical maltreatment	.27 (.88)	.40 (1.02)	.65 (1.21)	.67 (1.34)	.40*	.18*	.33*	.20*	1.00	
6. Paternal physical maltreatment	.18 (.73)	.32 (.95)	.54 (1.30)	.90 (1.51)	.24*	.39*	.22*	.26*	.30*	1.00
7. Sexual maltreatment	.51 (1.30)	1.55 (2.02)	1.37 (2.05)	2.65 (2.29)	.30*	.24*	.34*	.22*	.21*	.27*

* *p* < .01

Parameter estimates for the multinomial logistic model are given in Table 3. The overall model was significant, χ^2^(21, *N* = 720) = 135.14, *p* < .001. Higher severity of paternal emotional maltreatment was associated with a significantly greater likelihood of sexual revictimization only. Specifically, for every one standard deviation increase in the paternal emotional maltreatment score, the odds of experiencing severe sexual revictimization increased by 42%. Paternal physical abuse severity was significantly associated with a greater likelihood of sexual + physical revictimization. For every one standard deviation increase in physical abuse, there was a 29% increase in the odds of experiencing sexual + physical revictimization. Higher severity of sexual maltreatment was associated with a significantly higher likelihood of severe sexual and/or physical revictimization over no revictimization. For every one standard deviation increase in the sexual maltreatment score, the odds of experiencing severe sexual, physical, or sexual + physical revictimization increased by 92%, 74%, and 163%, respectively. See Supplemental Appendix B for descriptive characteristics of the sexual abuse severity indicators.

**Table 3.** Parameter Estimates for the Relations of Childhood Maltreatment Types to Severe Revictimization Groups.

	Wald	Exp (*B*)	*p*	95% CI for Exp(*B*)
Sexual only versus none				
Maternal emotional maltreatment	0.031	1.03	.86	[0.77, 1.37]
Paternal emotional maltreatment	5.86	1.42	.02	[1.07, 1.88]
Maternal neglect	0.95	1.17	.33	[0.86, 1.58]
Paternal neglect	3.94	0.75	.05	[0.56, 1.00]
Maternal physical maltreatment	0.03	1.02	.86	[0.83, 1.26]
Paternal physical maltreatment	0.12	0.96	.73	[0.77, 1.21]
Sexual maltreatment	36.33	1.92	.001	[1.55, 2.37]
Physical only versus none				
Maternal emotional maltreatment	0.05	0.95	.83	[0.57, 1.57]
Paternal emotional maltreatment	0.12	0.91	.73	[0.55, 1.52]
Maternal neglect	0.02	0.96	.88	[0.57, 1.63]
Paternal neglect	0.03	1.04	.87	[0.64, 1.69]
Maternal physical maltreatment	2.36	1.27	.12	[0.94, 1.71]
Paternal physical maltreatment	2.20	1.27	.14	[0.93, 1.73]
Sexual maltreatment	10.84	1.74	.001	[1.25, 2.42]
Sexual + physical versus none				
Maternal emotional maltreatment	0.002	1.01	.96	[0.67, 1.52]
Paternal emotional maltreatment	1.20	1.25	.27	[0.84, 1.85]
Maternal neglect	1.26	1.27	.26	[0.84, 1.94]
Paternal neglect	0.41	0.88	.52	[0.59, 1.31]
Maternal physical maltreatment	0.05	1.03	.83	[0.79, 1.34]
Paternal physical maltreatment	4.10	1.29	.04	[1.01, 1.65]
Sexual maltreatment	52.59	2.63	.001	[2.02, 3.41]

### Multivariate Relations of Childhood Maltreatment Types to Sexual Revictimization Among Plurisexual Women

Ninety-five women in the sample identified as plurisexual. To account for the relatively small size of this subgroup, the emotional maltreatment and neglect variables were combined, resulting in five maltreatment variables entered as a block into a multivariate general linear model (maternal emotional maltreatment/neglect, paternal emotional maltreatment/neglect, maternal physical maltreatment, paternal physical maltreatment, sexual maltreatment) and examined in relation to the presence versus absence of severe sexual revictimization.

The overall model was significant, *F*(1, 94) = 2.73, *p* = .02, Wilk’s Λ = 0.87, partial η^2^ = .13. Consistent with the full sample, higher scores on the sexual maltreatment scale were significantly associated with greater risk for sexual revictimization, (*B* = 0.66, SE = 0.23, *p* = .01, partial η^2^ = .07). Specifically, among plurisexual women, every one standard deviation increase in the sexual maltreatment score was associated with a 66% increase in likelihood of sexual re-revictimization. However, in direct contrast to the full sample, *maternal* emotional maltreatment/neglect (*B* = 0.66, SE =0.21, *p* = .002, partial η^2^ = .10) and *maternal* physical maltreatment (*B* = 0.54, SE = 0.21, *p* = .03, partial η^2^ = .05) were significantly associated with adult sexual revictimization, whereas paternal emotional maltreatment/neglect (*B* = 0.22, SE = 0.21, *p* = .29, partial η^2^ = .01) and paternal physical maltreatment (*B* = 0.40, SE = .21, *p* = .07, partial η^2^ = .03) were not. Among plurisexual women, every one standard deviation increase in maternal emotional maltreatment/neglect and maternal physical maltreatment was associated with a 66% and 54%, respectively, increase in the likelihood of reporting adult sexual re-revictimization.

## Discussion

The current study provided a fine-grained examination of the differential associations of several types of childhood maltreatment and in a novel extension of the literature examined risk for both sexual and/or physical revictimization in adulthood. Consistent with previous research, childhood sexual maltreatment was significantly associated with adulthood revictimization, and this relation was independent of the contributions of the other types of maltreatment. Further, consistent with hypotheses, emotional and physical maltreatment specifically perpetrated by the father were significantly and independently associated with sexual revictimization, whereas maltreatment perpetrated by the mother was not. In direct contrast, we provide novel evidence that among women who identified as plurisexual, it was maltreatment perpetrated by the mother that significantly and independently predicted risk for sexual revictimization.

### Childhood Sexual Maltreatment and Adult Revictimization

In a multivariate model that entered all forms of maltreatment simultaneously to account for their overlap, sexual maltreatment emerged with the largest effect size in association with adulthood sexual and physical revictimization. Among plurisexual women, childhood sexual maltreatment again emerged as having the strongest association with sexual revictimization, suggesting that sexual maltreatment is a general risk factor cutting across demographic groups.

The clinical significance of sexual maltreatment’s impact over other forms on risk for revictimization is indicated by the strength of the effects. Notably, sexual maltreatment evidenced at least two times greater predictive power than all other maltreatment types in its association with sexual revictimization on its own, and at least three times the predictive power than all other maltreatment types in its association with sexual + physical revictimization. Indeed, the sexual maltreatment scores of women in the sexual + physical revictimization group exceeded by a factor of 2.5 the cut-off for “severe” sexual maltreatment identified by Bifulco et al. (2005). These results are consistent with betrayal trauma theory (Freyd et al., 2007) in emphasizing the especially impactful consequences of sexual maltreatment, and they extend it to suggest that this risk goes beyond sexual revictimization to physical forms of interpersonal violence and assault. Therefore, these results highlight the need to target specific mechanisms related to the psychobiological sequelae and socio-environmental contexts of sexual maltreatment over and above other types of maltreatment, in developing strategies to prevent revictimization.

As suggested by Freyd et al. (2007), childhood sexual maltreatment may have unique and enduring sequelae that affect interpersonal behavior throughout life. Therefore, an important future research direction suggested by the current results is to determine whether mechanisms that translate childhood sexual maltreatment to later sexual revictimization are distinct from, or overlapping with, the mechanisms that translate sexual maltreatment to later physical revictimization. For example, sexual maltreatment is significantly associated with risky sexual behaviors, such as a greater likelihood of engagement in sexual activity while under the influence of drugs/alcohol and lower sexual assertiveness (Negriff et al., 2015), even when histories of physical or emotional maltreatment are controlled for. Risky sexual behaviors are also strongly associated with risk for sexual revictimization (Scoglio et al., 2021), with number of partners and low sexual assertiveness mediating the relation between sexual maltreatment and sexual revictimization in adulthood (Santos-Iglesias & Sierra, 2012). There is also some preliminary evidence that risky sexual behaviors raise risk for physical assault (Moore et al., 2017). While beyond the scope of the current investigation, an intriguing question for future research is to determine the extent to which these risky sexual behaviors *differentially* mediate the relation of sexual maltreatment to sexual versus physical revictimization.

### Paternal Emotional and Physical Maltreatment and Adult Revictimization

Consistent with our hypotheses, in the full sample, emotional maltreatment perpetrated by the father, but not the mother, was significantly associated with greater risk of sexual revictimization in adulthood. Further, physical maltreatment perpetrated by the father, but not the mother, was significantly associated with greater risk of both sexual *and* physical revictimization. Notably, paternal emotional maltreatment was associated with a 14-fold greater risk of sexual revictimization than was maternal emotional maltreatment (42% vs. 3%), and paternal physical maltreatment was associated with a 10-fold greater risk of sexual + physical revictimization than was maternal physical maltreatment (29% vs. 3%).

The specific relation of hostility, criticism, and violence by the father and women’s risk for sexual and/or physical victimization in their adult relationships is consistent with attachment theory, and with literature suggesting that the quality of the father–daughter relationship forms the basis upon which women’s relational scripts with men are developed (Cunningham et al., 2019). For example, women who have more positive father–daughter relationships have lower incidences of sexual assault after the age of 16 (Jankowski et al., 2002), are more confident in refusing unwanted sexual advances, and have less acceptance of male-dominance dating scripts (Katz & van der Kloet, 2010). Moreover, physical maltreatment perpetrated by the father is associated with higher levels of fear (Hamby et al., 2013) than that perpetrated by the mother, potentially driving the emergence of psychopathology that interferes with successful relationship functioning in adulthood. Future research should also consider examining potential interaction effects between sexual maltreatment and parental emotional maltreatment given the moderating effect parental reactions to sexual maltreatment disclosure has on psychopathology development (Hakimi et al., 2018). However, large sample sizes will be required to examine potential additive and interactive relations between the various forms of maltreatment.

### Maternal Maltreatment and Sexual Revictimization in Plurisexual Women

In the sample of women identifying as plurisexual it was maltreatment perpetrated by the *mother*, not the father, that was significantly associated with adult sexual revictimization. This novel finding should be replicated before firm conclusions are drawn. Nevertheless, these results tentatively suggest a stronger role for the mother–daughter relationship over the father–daughter relationship in driving sexual outcomes in this subgroup of women. The reasons for this differential pattern go beyond the current investigation and require further research. Consistent with the discussion above, it is possible factors such as attachment to mother versus father figures may differentially mediate the relation of maternal- versus paternal-perpetrated maltreatment to later sexual victimization in plurisexual versus monosexual women.

Consistent with a growing body of research (Flanders et al., 2019; Sterzing et al., 2019), women in the current sample who identified as plurisexual were 50% more likely to report exposure to severe sexual victimization in adulthood than monosexual women (61% of women in the plurisexual group reported exposure to sexual victimization vs. 42% of those in the monosexual group). Therefore, plurisexual individuals may also have unique risk factors for sexual victimization even when compared to other sexual minorities, including stereotypes of hypersexuality and assumptions of automatic consent (Flanders et al., 2019). Interestingly, heterosexism in general has not been associated with risk for sexual victimization, suggesting that risk is conferred by bi/plurisexual-specific stigma (Flanders et al., 2017). An important direction for future research is to determine how intersecting gender and ethnic minority identities contribute additive or interactive risk for sexual victimization in plurisexual individuals.

### Limitations

The current results should be interpreted in light of the following limitations. First, our study included predominantly cisgender, heterosexual, White women. Therefore, replication of these results in a more diverse sample is needed, and future studies are required to examine important differences in the relation between childhood maltreatment and adult revictimization across these intersecting diverse identities. Second, neglect and emotional and physical maltreatment were situated within the intrafamilial context whereas sexual maltreatment could be by any perpetrator. Future work should examine the potentially differential effects of intra- versus extrafamilial maltreatment on later revictimization. Third, the current investigation relied on cross-sectional, retrospective self-reports, which limits our ability to make conclusions about the temporal ordering, and causal associations, between our variables and introduces potential confounds related to recall bias. Our study attempted to address these concerns in several ways. We assessed childhood maltreatment and revictimization in two completely separate “studies” to minimize the chance that participants’ responses on the CECA-Q would bias responses on the SAPA, or vice versa. Further, we collected contextual information regarding the perpetrator(s), frequency, and severity of the maltreatment and revictimization exposures, and we independently categorized revictimization exposures using criteria developed and standardized by Bifulco et al. (1994). Indeed, one strength of our study was our measures of both childhood and adulthood victimization followed the same contextual approach. As such, the rating system was developed based on similar criteria, thereby maximizing statistical power. We also inspected each individual record and excluded participants whose reports on the CECA-Q and SAPA overlapped; that is, they were reporting on chronic victimization by the same perpetrator from childhood into adulthood. Nevertheless, prospective, longitudinal designs are required to clarify the distinct developmental pathways from types of maltreatment in childhood to revictimization in adulthood. Fourth, this study was administered through an online recruitment platform, raising concerns about data quality. We conducted validity checks within and between the two online questionnaires and used Prolific Academic, which specializes in behavioral science research and has been shown to include participants who devote more attention to questions and answer questions more accurately and honestly than the participants in other recruitment platforms (Peer et al., 2021). Nevertheless, replication in an epidemiological sample is required.

## Conclusion

Childhood maltreatment has negative, often lifelong, effects on functioning, and a primary mechanism through which it exerts its devastating impact is by raising risk for sexual and/or physical victimization throughout adulthood. The current results confirm the strong relation of childhood sexual abuse to risk for both sexual *and* physical revictimization in adulthood. Further, we provide novel evidence for the differential impact of emotional and physical abuse perpetrated by the father versus the mother on revictimization risk and suggest that this association may be expressed differently in women of differing sexual identities. These results have important implications for identifying girls at highest risk for revictimization and providing them with targeted support and intervention tailored to preventing this risk. Relatedly, a crucial next step is to determine the psychological, biological, and sociocultural mediators and moderators of the relation between particular types of childhood maltreatment and sexual and physical revictimization. Identifying modifiable targets will enable the development of specific intervention programs that have the potential for maximum efficacy in preventing revictimization. Given the overwhelming personal and economic costs associated with maltreatment and victimization, these future directions should be a pressing public health priority.

## Supplemental Material

sj-docx-1-jiv-10.1177_08862605221111411 – Supplemental material for Sexual, Physical, and Emotional Maltreatment in Childhood Are Differentially Associated With Sexual and Physical Revictimization in Adulthood Supplemental material, sj-docx-1-jiv-10.1177_08862605221111411 for Sexual, Physical, and Emotional Maltreatment in Childhood Are Differentially Associated With Sexual and Physical Revictimization in Adulthood by Jessica Rowe, Jasmine Chananna, Simone Cunningham and Kate L. Harkness in Journal of Interpersonal Violence

sj-docx-2-jiv-10.1177_08862605221111411 – Supplemental material for Sexual, Physical, and Emotional Maltreatment in Childhood Are Differentially Associated With Sexual and Physical Revictimization in Adulthood Supplemental material, sj-docx-2-jiv-10.1177_08862605221111411 for Sexual, Physical, and Emotional Maltreatment in Childhood Are Differentially Associated With Sexual and Physical Revictimization in Adulthood by Jessica Rowe, Jasmine Chananna, Simone Cunningham and Kate L. Harkness in Journal of Interpersonal Violence
