# Supplementary material for: Sexual, Physical, and Emotional Maltreatment in Childhood Are Differentially Associated With Sexual and Physical Revictimization in Adulthood
Source: J Interpers Violence. 2022 Jul 22;38(3-4):3806–30. doi: 10.1177/08862605221111411 (PMC9850393; doi:10.1177/08862605221111411)
Supplement: sj-docx-1-jiv-10.1177_08862605221111411 – Supplemental material for Sexual, Physical, and Emotional Maltreatment in Childhood Are Differentially Associated With Sexual and Physical Revictimization in Adulthood [file sj-docx-1-jiv-10.1177_08862605221111411.docx]

**Appendix A: Sexual and Physical Assault Scale (SAPA)**

| Table B.1 *Sexual Assault Scale* |  |
| --- | --- |
| Since the age of 17 has anyone forced you or persuaded you to have a sexual experience, even though you were not willing, that included… |  |
| 1. Kissing and/or petting 2. The other person touching your genitals 3. Touching the other person’s genitals 4. Vaginal, oral, or anal intercourse 5. Verbal threats towards you or someone else 6. Physical force or violence   For each yes (Y) above indicate   1. How often has this happened since age 17    1. Once only    2. 2-4 Times    3. 5-9 Times    4. 10+ Times 2. Who was the other person (select all that apply)    1. Spouse    2. Close family member (parent, sibling)    3. Other relative (grandparent, aunt/uncle, cousin)    4. Boyfriend/girlfriend    5. Friend/Acquaintance    6. Authority figure (e.g., doctor, priest/rabbi, teacher)    7. Stranger    8. Other | (Y/N)  (Y/N)  (Y/N)  (Y/N)  (Y/N)  (Y/N) |

| Table B.2 *Physical Assault Scale* |  |
| --- | --- |
| Since the age of 17, have you ever had an experience of non-sexual physical assault that included... |  |
| 1. Slapping or pinching 2. Punching or kicking the arms, legs, or body 3. Punching or kicking the head or face 4. Hitting with an object (e.g., belt, stick) 5. Threatening with a knife, gun, or other object? 6. Bruising, black eyes, surface cuts 7. Broken bones, concussion, or other serious injury (e.g., burns, deep wounds)   For each yes (Y) above indicate   1. How often has this happened since age 17    1. Once only    2. 2-4 Times    3. 5-9 Times    4. 10+ Times 2. Who was the other person (select all that apply)    1. Spouse    2. Close family member (parent, sibling)    3. Other relative (grandparent, aunt/uncle, cousin)    4. Boyfriend/girlfriend    5. Friend/Acquaintance    6. Authority figure (e.g., doctor, priest/rabbi, teacher)    7. Stranger    8. Other | (Y/N)  (Y/N)  (Y/N)  (Y/N)  (Y/N)  (Y/N)  (Y/N) |
