# Supplementary material for: Sexual, Physical, and Emotional Maltreatment in Childhood Are Differentially Associated With Sexual and Physical Revictimization in Adulthood
Source: J Interpers Violence. 2022 Jul 22;38(3-4):3806–30. doi: 10.1177/08862605221111411 (PMC9850393; doi:10.1177/08862605221111411)
Supplement: sj-docx-2-jiv-10.1177_08862605221111411 – Supplemental material for Sexual, Physical, and Emotional Maltreatment in Childhood Are Differentially Associated With Sexual and Physical Revictimization in Adulthood [file sj-docx-2-jiv-10.1177_08862605221111411.docx]

**Appendix B: Descriptive Characteristics of Sexual Maltreatment Severity Indicators**

Among the 268 women who reported any history of sexual maltreatment in childhood, we conducted follow-up regression analyses to determine if specific indicators of the sexual maltreatment were preferentially associated with later re-victimization. As seen in Table C.1, age at first incident of sexual maltreatment did not significantly predict risk for sexual and/or physical re-victimization, and neither did relation to the perpetrator. Further, exposure to multiple versus only one incident of sexual maltreatment predicted greater risk for severe physical re-victimization only. Further, sexual maltreatment involving intercourse versus no intercourse predicted greater risk for sexual+physical re-victimization at a trend (*X^2^* (2, 268) *= 5.67, p = 0.06*).

| Table C.1 *Sexual Abuse Severity Indicators Stratified by Adulthood Revictimization Among Participants Who Reported Sexual Abuse (n = 268)* | | | | | |
| --- | --- | --- | --- | --- | --- |
| Characteristics of Sexual Abuse | No Victimization  (*n* = 63) | Sexual Victimization (*n =* 121) | Physical Victimization  (*n* = 19) | Sexual + Physical Victimization  (*n* = 65) | *X^2^*/*F* |
| Age at first incident, *M*(*SD*) | 11.95(4.19)_a_ | 12.02(4.71)_a_ | 10.11(4.08)_a_ | 11.64(4.64)_a_ | 1.03 |
| Relation to Perpetrator, *n*(%) |  |  |  |  | 5.70 |
| Relative household member | 8(19.0)_a_ | 20(47.6)_a_ | 2(4.8)_a_ | 12(28.6)_a_ |  |
| Relative non-household member | 10(25.6)_a_ | 12(30.8)_a_ | 4(10.3)_a_ | 13(33.3)_a_ |  |
| Non-relative | 45(24.1)_a_ | 89(47.6)_a_ | 13(7.0)_a_ | 40(21.4)_a_ |  |
| Frequency, *n*(%) |  |  |  |  | 10.10* |
| One incident | 39(28.3)_a_ | 60(43.5)_a_ | 4(2.9)_b_ | 35(25.4)_a_ |  |
| Multiple incidents | 24(18.5)_a_ | 61(46.9)_a_ | 15(11.5)_b_ | 30(23.1)_a_ |  |
| Intensity, *n*(*%*) |  |  |  |  | 10.46 |
| No genital touching/intercourse | 10(26.3)_a_ | 15(39.5)_a_ | 5(13.2)_a_ | 8(21.1)_a_ |  |
| Genital touching | 40(25.8)_a_ | 76(49.0)_a_ | 8(5.2)_a_ | 31(20.0)_a_ |  |
| Sexual intercourse | 13(17.3)_a_ | 30(40.0)_a_ | 6(8.0)_a_ | 26(34.7)_a_ |  |
| *Note:*  * *p <* .05  Means that do not share subscripts differ by *p* < .05 according to Tukey’s Honestly Significant Difference. Counts that do not share subscripts differ by *p* < .05 according to chi-square analyses. | | | | | |
